# Circulating miRNAs as Potential Biomarkers Associated with Cardiac Remodeling and Fibrosis in Chagas Disease Cardiomyopathy

**DOI:** 10.3390/ijms20164064

**Published:** 2019-08-20

**Authors:** Carolina Kymie Vasques Nonaka, Carolina Thé Macêdo, Bruno Raphael Ribeiro Cavalcante, Adriano Costa de Alcântara, Daniela Nascimento Silva, Milena da Rocha Bezerra, Alex Cleber Improta Caria, Fábio Rocha Fernandes Tavora, João David de Souza Neto, Márcia Maria Noya-Rabelo, Silvia Regina Rogatto, Ricardo Ribeiro dos Santos, Bruno Solano de Freitas Souza, Milena Botelho Pereira Soares

**Affiliations:** 1 Center for Biotechnology and Cell Therapy, Hospital São Rafael, 41253-190 Salvador, Brazil; 2 Gonçalo Moniz Institute, FIOCRUZ, 40296-710 Salvador, Brazil; 3 D’Or Institute for Research and Education (IDOR), 22281-100 Rio de Janeiro, Brazil; 4 Department of Cardiology, São Rafael Hospital, 41253-190 Salvador, Brazil; 5 Federal University of Bahia, UFBA, 40231-300 Salvador, Brazil; 6 Messejana Hospital, 60846-190 Fortaleza, Brazil; 7 Department of Clinical Genetics, Vejle Hospital, Institute of Regional Health Research, University of Southern Denmark, 7100 Vejle, Denmark; 8 National Institute of Science and Technology for Regenerative Medicine, 21941-902 Rio de Janeiro, Brazil

**Keywords:** chagas disease, cardiomyopathy, microRNA, fibrosis, inflammation

## Abstract

Chagas disease (CD) affects approximately 6–7 million people worldwide, from which 30% develop chronic Chagas cardiomyopathy (CCC), usually after being asymptomatic for years. Currently available diagnostic methods are capable of adequately identifying infected patients, but do not provide information regarding the individual risk of developing the most severe form of the disease. The identification of biomarkers that predict the progression from asymptomatic or indeterminate form to CCC, may guide early implementation of pharmacological therapy. Here, six circulating microRNAs (miR-19a-3p, miR-21-5p, miR-29b-3p, miR-30a-5p, miR-199b-5p and miR-208a-3p) were evaluated and compared among patients with CCC (*n* = 28), CD indeterminate form (*n* = 10) and healthy controls (*n* = 10). MiR-19a-3p, miR-21-5p, and miR-29b-3p were differentially expressed in CCC patients when compared to indeterminate form, showing a positive correlation with cardiac dysfunction, functional class, and fibrosis, and a negative correlation with ejection fraction and left ventricular strain. Cardiac tissue analysis confirmed increased expression of microRNAs in CCC patients. In vitro studies using human cells indicated the involvement of these microRNAs in the processes of cardiac hypertrophy and fibrosis. Our study suggests that miRNAs are involved in the process of cardiac fibrosis and remodeling presented in CD and indicate a group of miRNAs as potential biomarkers of disease progression in CCC.

## 1. Introduction

Chagas disease, caused by the protozoan parasite *Trypanosoma cruzi*, affects approximately 6–7 million people worldwide mainly in Latin American countries, and is spreading to other continents due to population migration [1]. The majority of infected patients will remain asymptomatic during the chronic phase, in what is known as the indeterminate form of Chagas disease, while approximately 30% will develop the cardiac form, also known as chronic Chagas disease Cardiomyopathy (CCC), with a high rate of morbidity and mortality [2]. Residual parasitism is found in different organs in chronic Chagas disease, despite the clinical presentation [3]. In the cardiac form, however, myocardial dysfunction occurs due to parasite persistence, chronic inflammation [4,5], and cardiac fibrosis [6]. Additionally, cardiac remodeling can lead to heart chamber dilation, wall thinning, arrhythmia, and cardiac hypertrophy [4,7,8,9]. 

Although currently available diagnostic tests are capable of adequately identifying infected patients, there is no biomarker that can predict the individual risk for a patient’s potential to progress from the indeterminate form to CCC [8]. Therefore, the identification of novel biomarkers allowing for early identification of myocardial damage is desirable for interventions aiming at delaying heart dysfunction development and progression. 

MicroRNAs are a class of non-coding RNAs with approximately 22 nucleotides that are widely distributed and frequently conserved among species, and act through regulating gene expression at the post-transcriptional level [10,11,12,13]. Functional microRNAs can be detected in whole blood, serum, and plasma, with potential application as diagnostic or prognostic biomarkers [11,12,13,14]. Altered expression levels of circulating microRNAs in different body fluids have been reported in several disease settings [13,14,15], including cardiovascular diseases [16,17]. In this context, studies demonstrated that miR-19a contributes to heart failure [18] whereas miR-21 [19], miR-29 [20,21], and the miR-30 family [22] contribute to cardiac fibrosis or hypertrophy, while miR-199b acts as a regulator of left ventricular remodeling, associated with cardiac hypertrophy [23].

In Chagas disease, the expression levels of miR-208a and miR-208b were altered in the heart from CCC subjects [24]. Additionally, the potential of miR-208a as a biomarker for Chagas disease has been explored [25]. In the present study, we evaluated the expression profile of circulating microRNAs in three groups: subjects with CCC, subjects with indeterminate form of Chagas disease, and healthy controls, in order to identify possible correlations with clinical parameters associated with the disease prognosis. Additionally, we compared the expression of selected microRNAs in CCC hearts, and explored their roles in vitro using human cardiac fibroblasts and cardiomyocytes.

## 2. Results

### 2.1. Expression Profile of Circulating microRNAs in Subjects with Chronic Chagas Disease

Since the detection of circulating miRNAs can vary within each type of analyzed sample [26], we compared the levels of the selected miRNAs in serum and plasma samples obtained from 16 chronic Chagas subjects. Increased expression levels of miR-19a-3p, miR-29b-3p, and miR-30a-5p were observed in serum samples when compared to plasma from the same subjects (Figure S1A,C,D). No differences were observed between miR-21-5p or miR-199b-5p when comparing serum and plasma samples (Figure S1B,E). Amplification of miR-208a-3p was successful in only four samples, with higher levels detected in plasma samples (Figure S1F). 

The following validation analysis was performed in serum samples from 42 subjects, stratified into three groups (healthy, indeterminate form, and CCC). Patients with Chagas disease underwent evaluations by echography and MRI. Clinical and demographic characteristics of the Chagas disease patients are shown in Table 1. 

**Figure 1:**
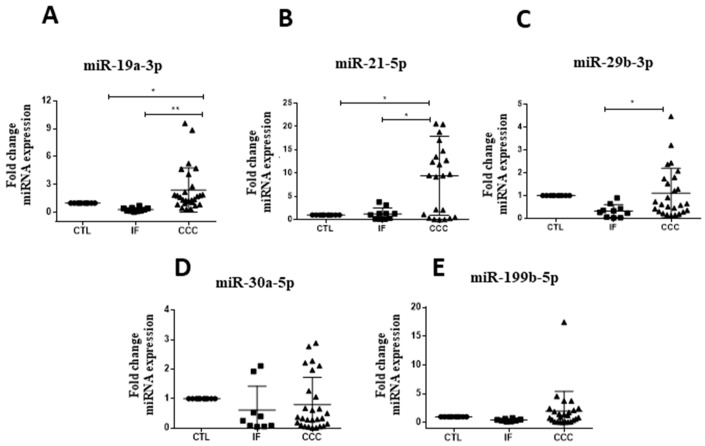
MicroRNA validation in serum of chronic Chagas disease subjects and healthy controls. Healthy controls (CTL), *n* = 10, Indeterminate form (IF), *n* = 10, and chronic Chagas cardiomyopathy (CCC), *n* = 28, were evaluated by RT-qPCR, (**A**) miR-19a-3p, (**B**) miR-21-5p and (**C**) miR-29b-3p, (**D**) miR-30a-5p and (**E**) miR-199b-5p, normalized by miR-93 and miR-16. One-way ANOVA followed by Newman-Keuls post-test. * *p* < 0.05.

Upregulation of miR-19a-3p, miR-21-5p, and miR-29b-3p were observed in subjects with CCC when compared to Chagas disease indeterminate form (Figure 1). For all miRNAs tested, similar levels were found between healthy controls and indeterminate form subjects. Expression levels of miR-30a-5p and miR-199b-5p were similar between the tested groups (Figure 1A–E). MiR-208a-3p was not detected in the majority of the samples evaluated.

**Figure 2:**
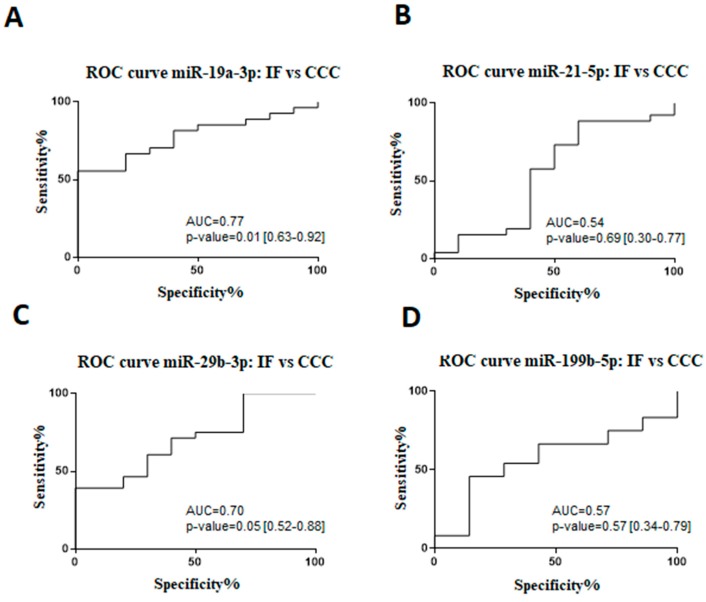
Receiver operating characteristic (ROC) curve for the prognosticating Chagas disease progression. ROC analysis of miRNA expression in serum from chronic Chagas disease subjects, indeterminate form (IF) vs chronic Chagas cardiomyopathy (CCC): (**A**) miR-19a-3p, (**B**) miR-21-5p, (**C**) miR-29, and (**D**) miR-199b-5p expression, measured by RT-qPCR. The miRNA values were standardized for statistical analysis. The area under the curve (AUC) with 95% CI and P value were represented in the figure. *p* < 0.05 indicate statistical significance.

A receiver operating characteristic (ROC) analysis was performed to evaluate sensitivity, specificity, and accuracy of the miR-19a-3p, miR-21-5p, miR-29b-3p, and miR-199b-5p as cardiac dysfunction biomarkers in Chagas disease patients (Figure 2). By comparing indeterminate and CCC groups, it was demonstrated that the area under the curve (AUC) for miR-19a-3p was 0.77 (67% sensitivity and 80% specificity), miR-21-5p was 0.54 (57% sensitivity and 60% specificity), miR-29b-3p was 0.70 (60% sensitivity and 70% specificity), and miR-199b-5p was 0.57 (67% sensitivity and 57% specificity).

Next, we evaluated the correlation between clinical parameters of Chagas disease (indeterminate vs CCC) and the expression levels of the selected miRNAs. MiR-19a-3p and miR-21-5p expression levels showed a positive correlation with cardiac dysfunction, functional class (NYHA II/IV), and percentage of fibrosis measured by cardiac MRI. Negative correlations were found between the levels of miR-19a-3p and miR-21-5p ejection fraction and left ventricular strain, measured by echocardiography. Furthermore, miR-29b-3p was negatively correlated with ejection fraction and positively correlated with cardiac dysfunction, functional class, and percentage of fibrosis (Table 2). 

### 2.2. MicroRNAs in Cardiac Tissue Samples of Subjects with End-Stage CCC

**Figure 3:**
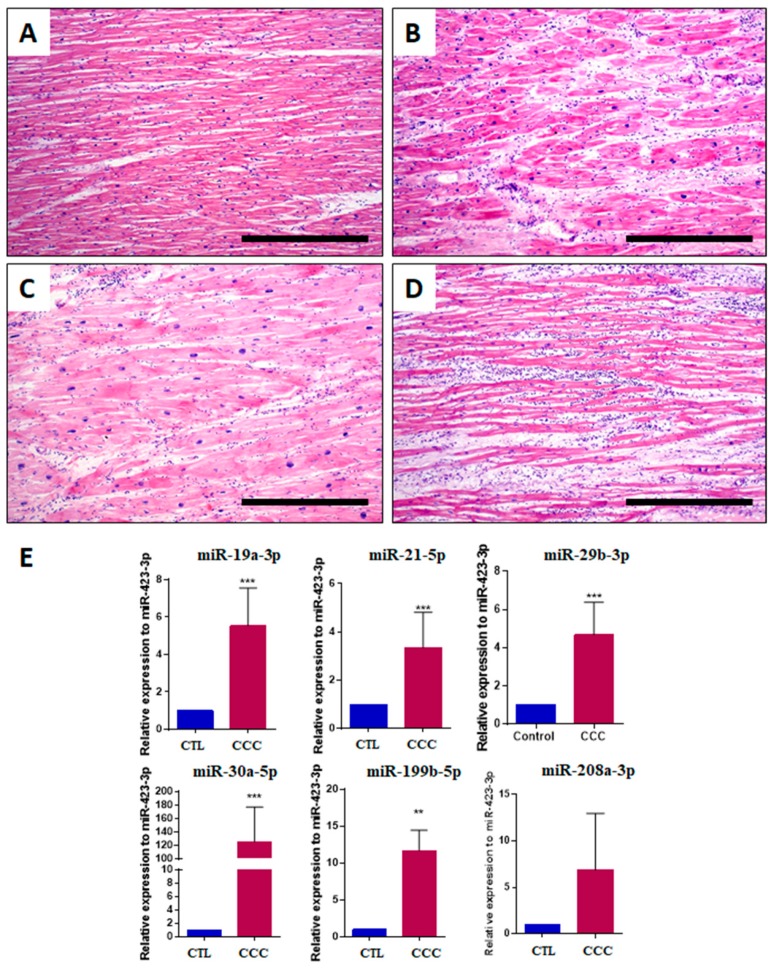
Histological and miRNA analysis in explanted hearts from chronic Chagas disease subjects. Left ventricle sections stained with H&E showing (**A**) a normal area of myocardium with minimal myocyte hypertrophy; (**B**) interstitial fibrosis and moderate myocyte hypertrophy; (**C**) severe myocyte hypertrophy, with the presence of enlarged and pleomorphic nuclei, varying in shape and size. The fibers are random and there is focal interstitial inflammation (top left corner); (**D**) multifocal inflammation characterized by accumulation of plasma cells and mature lymphocytes. The nuclei of myocytes are mildly enlarged and pleomorphic (moderate myocyte hypertrophy), and there is mild interstitial fibrosis. Bars = 500 μm; (**E**) miR-19a-3p, miR-21-5p, miR-29b-3p, miR-30a-5p, miR-199b-5p and miR-208a-3p evaluation by RT-qPCR, using 2^−∆∆Ct^ method and normalized with miR-423-3p. Data represent the mean ± SEM. Controls (CTL), *n* = 8, and chronic Chagas cardiomyopathy (CCC), *n* = 9, analyzed using Student’s *t*-test, *** *p* < 0.001, ** *p* < 0.01.

Left ventricular sections obtained from explanted hearts were evaluated after staining with hematoxylin and eosin, showing the presence of inflammatory infiltrates composed of mononuclear cells, areas of cardiac fibrosis and hypertrophic myocytes (Figure 3A–D). In order to evaluate whether the microRNA expression in the cardiac tissues is consistent with the findings detected in the serum, explanted cardiac tissues from chronic Chagas disease subjects (*n* = 9) and unaffected controls (*n* = 8) were evaluated by RT-qPCR. Increased expression levels of all microRNAs tested (miR-19a-3p, miR-21-5p, miR-29b-3p, miR-30a-5p, miR-199b-5p and miR-208a-3p) were found in the heart samples from subjects with Chagas disease when compared to controls (Figure 3E).

### 2.3. MicroRNAs Expression Levels in Fibrosis Using Human Cardiac Fibroblasts

**Figure 4:**
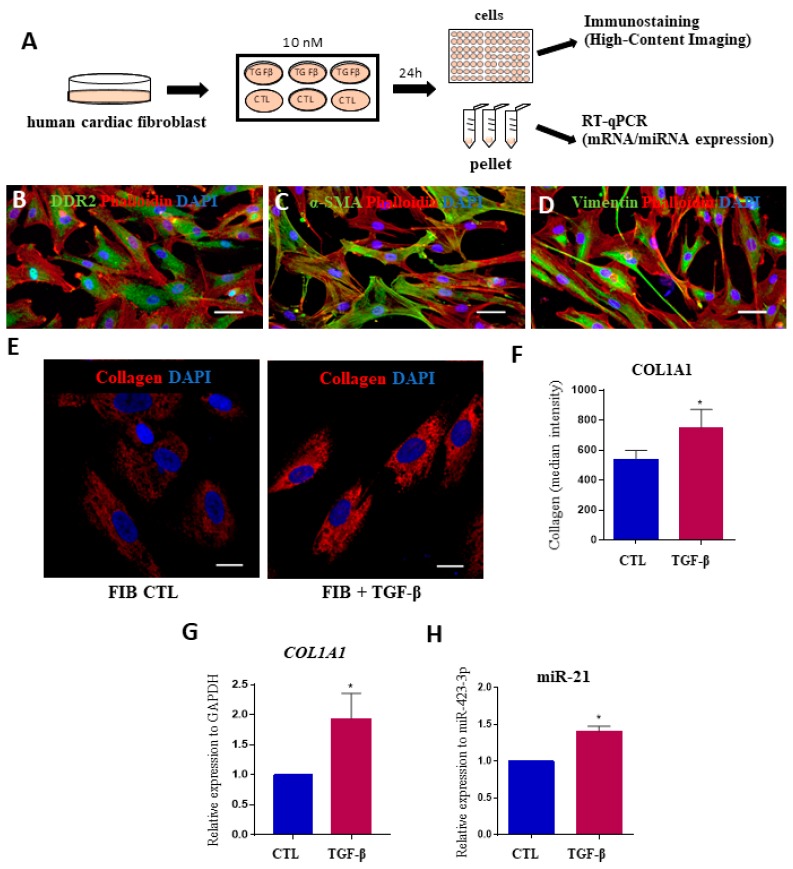
Assessment of miRNA expression in human cardiac fibroblasts after stimulation with TGFβ. (**A**) Experimental design. (**B**) Validation of human cardiac fibroblasts markers by immunostaining for DDR2 (**A**, green), α-SMA (**B**, green) and vimentin (**C**, green). In all samples, the cytoskeleton was stained with phalloidin (red) and nuclei were stained with DAPI (blue). Scale bars = 50 μm; (**E**) Staining for collagen type I (red) and nuclei were stained with DAPI (blue) after 24 h of incubation with TGF-β. Scale bars = 20 μm; (**F**) Quantification of COL1A1 expression by measurements of median fluorescence intensity and (**G**) gene expression by RT-qPCR. (**H**) Expression levels of miR-21-5p in human cardiac fibroblasts stimulated or not with TGF-β1, evaluated by RT-qPCR, normalized with miR-423-3p and represented by fold change expression. CTL = unstimulated cardiac fibroblasts. TGF-β = cardiac fibroblasts stimulated with TGF-β. Values represent the mean ± SEM of n=3 replicates group and were evaluated using Student’s *t*-test. * *p* < 0.05.

To evaluate the possible involvement of miRNAs in the cardiac fibrosis process that occurs in Chagas disease, we used an in vitro model of human cardiac fibroblasts stimulated with the profibrogenic factor TGF-β1. This cytokine found in highly elevated levels in CCC [27] and induces collagen production (Figure 4A). First, markers of fibroblasts, such as DDR2, αSMA, and vimentin, were detected in cells obtained from heart explants (Figure 4B–D). TGF-β1 stimulation was associated with a significant increase in *COL1A1* expression, as shown by high-content imaging and RT-qPCR analyses (Figure 4E–G). Overexpression of miR-21-5p was found in TGF-β1-stimulated cardiac fibroblasts, compared to unstimulated cells (Figure 4H). Other miRNAs tested (miR-19a-3p, miR-29b-3p, miR-30a-5p, miR-199b-5p and miR-208a-3p) were not altered by the addition of TGF- β1 in cardiac fibroblast cultures.

### 2.4. Expression of MicroRNAs in a Hypertrophy Model Using hiPSC-Derived Cardiomyocytes

**Figure 5:**
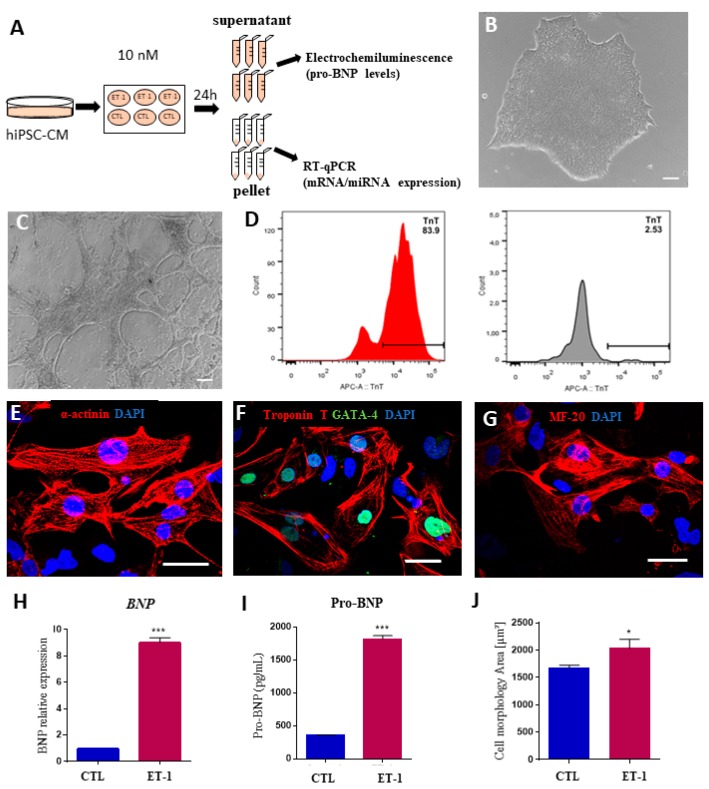
Validation of an in vitro hypertrophy model using human induced pluripotent stem cell-derived cardiomyocytes stimulated with endothelin-1. (**A**) Experimental design. (**B**–**G**) Characterization of hiPSC-CM. Morphology of hiPSCs visualized by phase-contrast microscopy at differentiation D0 (**B**) and D14 (**C**). (**D**) Differentiation efficiency measured by flow cytometry analysis of cTNT^+^ cells. Histograms show differentiated cells on the left (red histogram) and undifferentiated hiPSCs on the right (grey histogram). Scale bars = 100 μm. (**E**–**G**) Immunostaining for cardiomyocyte markers sarcomeric alpha-actinin (**E**), cardiac troponin T and GATA-4 (**F**), and MF-20 (**G**). Scale bars = 50 μm. (**H**) Increased BNP expression at the mRNA level, evaluated by RT-qPCR and at the protein level in the (**I**) culture supernatant 24 h after ET-1 incubation. (**J**) Cardiomyocyte area measured by quantification of cTNT staining. CTL = unstimulated hiPSC-CMs, ET1 = hiPSC-CMs stimulated with ET-1. Values represent the mean ± SEM of *n* = 3 replicates group and were evaluated using Student’s *t*-test. * *p* < 0.05 and *** *p* < 0.001.

Next, to investigate a possible role of the selected miRNAs in cardiomyocyte hypertrophy, a feature of CCC [28], we used an in vitro model based on human induced pluripotent stem cells-derived cardiomyocytes (hiPSC-CMs), stimulated with 10 nM ET-1 (Figure 5A). HiPSC-CMs presented spontaneous contractility and cardiac troponin T expression (Figure 5B–D). In addition, hiPSC-CMs were positively stained for the cardiomyocyte markers alpha-actinin, cardiac troponin T, GATA-4, and MF-20 (Figure 5E–G). Cardiomyocyte hypertrophy induction by ET-1 stimulation was confirmed by increased *NPPB* expression and NT-pro-BNP levels (Figure 5H–I). Moreover, ET-1-stimulated cardiomyocytes presented a significant increase in cell size, confirming a hypertrophic phenotype (Figure 5J). 

**Figure 6:**
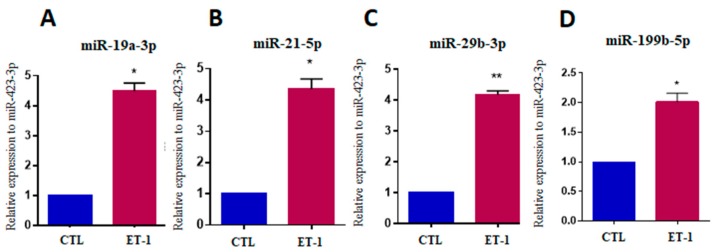
Increased miRNA expression after hypertrophic stimulus with endothelin-1 in hiPSC-CM. Expression levels of miR-19a-3p (**A**), miR-21-5p (**B**), miR-29b-3p (**C**) and miR-199b-5p (**D**) in hiPSC-CM stimulated with ET-1, evaluated by RT-qPCR. CTL = unstimulated hiPSC-CMs, ET1 = hiPSC-CMs stimulated with ET-1. Values represent the mean ± SEM of *n* = 3 per group. Data were analyzed using Student’s *t*-test. * *p* < 0.05; ** *p* < 0.01.

After validating the in vitro hypertrophy model, we evaluated the selected miRNAs. A significant increase in the expression of miR-19a-3p, miR-21-5p, miR-29b-3p and miR-199b-5p (*p* < 0.05) was observed in cardiomyocytes stimulated with ET-1 compared to untreated controls (Figure 6A–D).

## 3. Discussion

The potential role of miRNAs as novel biomarkers and therapeutic targets have been extensively investigated in the cardiovascular field [17]. Chagas disease, however, is a neglected disease, and, therefore, there are only two reports of miRNA expression in human samples, both focusing on cardiac tissue analyses [24,29]. The present study is the first to investigate the expression of circulating miRNAs - miR-19a-3p, miR-21-5p, miR-29b-3p, and miR-199b-5p in subjects with Chagas disease, and to perform correlation analyses with relevant clinical parameters, aiming at evaluating their potential role as biomarkers for cardiac dysfunction. 

Our results revealed that miR-19a-3p expression was higher in subjects with CCC compared to those with the indeterminate form and showed correlations with different clinical features of disease severity. Previously, miR-19a was associated with cardiomyocyte hypertrophy in vitro [30] and demonstrated increased cardiac expression in a mouse model of hypertrophic cardiomyopathy induced by aortic constriction [31]. These data corroborate the findings from our in vitro hypertrophy model with hiPSC-CM, which showed increased miR-19a-3p expression, along with BNP, and increased cardiomyocyte area after ET-1 exposure. MiR-19a was also found to be overexpressed in plasma of patients with heart failure [18] and was indicated as a potential novel biomarker for early diagnosis of acute myocardial infarction [32]. In the present study, miR-19a-3p expression revealed a positive correlation with the percentage of fibrosis and NYHA functional class, and a negative correlation with left ventricular strain and ejection fraction. Moreover, we found that miR-19a-3p was upregulated in the heart tissue of subjects with end-stage CCC, which presented typical histological characteristics of CCC: cardiomyocyte hypertrophy, chronic inflammation, and fibrosis [33]. These data suggest that miR-19a-3p could be further explored as a cardiac dysfunction progression marker in Chagas disease in a longitudinal study, especially focused on subjects in the indeterminate form, who may progress to a cardiac form.

In the present study, we found a negative correlation among serum miR-29b-3p expression and ejection fraction, and a positive correlation with cardiac fibrosis, NYHA functional, and cardiac dysfunction. We also demonstrated increased miR-29b-3p expression in heart samples from patients with end-stage CCC. Additionally, using an in vitro cardiomyocyte hypertrophy model, increased levels of miR-29b-3p were also observed. Similarly, a recent study reported that miR-29 promotes hypertrophic growth of cardiomyocytes and suggested that in vivo anti-miR-29 delivery might have therapeutic value for pathological cardiac remodeling and fibrosis [34].

The association between miR-21 and cardiac fibrosis in subjects with heart failure has been previously reported [35,36,37]. For the first time, we showed that miR-21-5p is also upregulated in the heart tissue of subjects with end-stage CCC. Additionally, our study confirmed that miR-21-5p is overexpressed in human cardiac fibroblasts following stimulation with TGFβ, a cytokine involved with heart fibrogenesis in different disease settings, including Chagas disease [27,38]. Furthermore, TGFβ signaling pathway has been demonstrated to be associated with miR-21 expression in fibrogenesis [39,40,41]. Interestingly, we also observed increased miR-21-5p in CCC subjects, when compared to indeterminate form subjects, positive correlation with cardiac dysfunction, NYHA class, and cardiac fibrosis, and negative correlation with ejection fraction and left ventricular. A previous report showed the association between miR-21 and hypertrophy in murine and human models during cardiac remodeling and heart failure [42]. These data corroborate our findings using an in vitro model of cardiomyocyte hypertrophy using hiPSC-CM. High miR-21 expression promotes cardiac hypertrophy and fibrosis and may be a key regulator in heart failure [36]. Evaluation of miRNA expression levels in hearts from *T. cruzi*-infected mice also demonstrated miR-21 upregulation in the acute phase of Chagas disease model [43].

Calcineurin/NFAT signaling is apparently regulated by the expression levels of miR-199b-5p in cardiac remodeling and dysfunction [23]. In our study, circulating miR-199b-5p levels were not increased in Chagas disease patients, when compared to healthy controls. However, we found that miR-199b-5p was upregulated in the heart tissue of subjects with end-stage CCC, and in our in vitro model, suggesting its involvement with cardiomyocyte hypertrophy. Further studies with increased sample size are necessary to clarify these results.

MiR-208a, a heart-specific miRNA, was correlated with the severity of coronary heart disease [44]. In our study, miR-208a-3p was not detected in most of the serum samples. A previous study reported upregulation of miR-208a-3p in plasma samples from patients with the indeterminate form of Chagas disease [25]. Based on these findings, a larger cohort comparing the expression levels of miR-208a-3p in plasma and serum would be relevant to better define the role of this miR and its value as a circulating biomarker. Previously, miR-208a was found to be upregulated in cardiac tissues obtained from patients with CCC [24]. In accordance, our study confirmed the expression of miR-208a previously described in heart samples from subjects with CCC.

Reinforcing our findings in serum samples, we observed increased expression of miRNAs in our in vitro models of cardiac fibrosis and hypertrophy. We found elevated expression of miR-19a-3p, miR-21-5p, miR-29b-3p, and miR-199b-5p in hiPSC-CM, whereas miR-21-5p was upregulated in cardiac fibroblasts. These results are in accordance with previous data showing altered miRNA expression in the same in vitro model of cardiac fibroblast [45] and cardiac hypertrophy using hiPSC-CM [46].

In our study, high expression levels of miR-29b-3p and miR-30a-5p were observed in explanted hearts of end-stage CCC patients. The involvement of a member of the miR-30 family in cardiomyocyte hypertrophy induction has been previously demonstrated [47], and miR-29 and miR-30 presented anti-fibrotic effects [48]. Additional studies are needed to better understand the roles of these miRNAs, since different miRNA expression patterns may reflect specific aspects of the complex pathogenesis and signaling pathways involved in the CCC.

It is well established that microRNAs can be detected in the blood [49], plasma [25], and serum [15]. However, a significant variation of the results could be observed, which may be due to different detection sensitivities of the circulating microRNAs measured in serum and plasma [11,26]. Nevertheless, in our study, the serum samples showed a higher miRNA concentration compared to plasma samples for most of the miRNAs evaluated. This difference in circulating miRNA concentrations in biofluids may be explained by the microRNA trafficking system between cellular compartments, extracellular environment, and coagulation process [26,50].

In conclusion, our results demonstrate alterations in the expression levels of circulating miRNAs in Chagas disease, some of which correlated with cardiac tissue expression profile and were shown to be regulated in cardiac fibroblasts and human cardiomyocytes, under pro-fibrotic and pro-hypertrophic conditions in vitro. Importantly, the increased circulating levels of miR-19a-3p, miR-21-5p, and miR-29b-3p correlated with cardiac injury and disease severity in Chagas disease. It is important to recognize that the present study is exploratory, with a small sample size and a cross-sectional design, in which a single time point measurement of the microRNAs was performed. These miRNAs could be further explored in a large cohort of cases as novel biomarkers or validated as molecular targets for therapeutic intervention.

## 4. Materials and Methods

### 4.1. Ethics and Study Design

This is an exploratory study consisting of the analysis of circulating microRNAs followed by heart tissue analysis and functional in vitro assays. The procedures complied with the Declaration of Helsinki and received prior approval by the Ethics Committee of the São Rafael Hospital (Protocol # 20023913.6.0000.0048, 07/30/2013), and has been registered at ClinicalTrials.gov under the identifier, NCT01842880. All subjects signed the written informed consent before inclusion in the study. Heart samples were obtained from the Heart Transplant Service at Messejana Hospital, Fortaleza, Brazil, with approval by the São Rafael Hospital’s Ethics committee (Protocol # 51025115.3.0000.0048).

### 4.2. Study Population

The circulating microRNAs were evaluated in 42 subjects divided into three groups: i. Chagas disease, cardiac form with left ventricular dysfunction, CCC (*n* = 28), ii. Chagas disease, indeterminate form (*n* = 10), and iii. healthy controls (*n* = 10). The participants were selected from a database of outpatient clinics from São Rafael Hospital (Table 1).

Subjects with CCC were included according to the following criteria: diagnosis of Chagas disease confirmed by two methods: indirect hemagglutination and indirect immunofluorescence, symptomatic heart failure (NYHA classes II, III and IV), left ventricular ejection fraction ≤55%, measured by Doppler echocardiogram - Simpson method, presence of myocardial fibrosis visualized as delayed enhancement in cardiac magnetic resonance imaging (MRI). Subjects with the indeterminate form of Chagas disease were selected using the following inclusion criteria: diagnosis of Chagas disease confirmed by indirect hemagglutination and indirect immunofluorescence; absence of clinical diagnosis of heart failure; absence of abnormalities in echography, Holter and MRI. The inclusion criteria for healthy controls were: age > 40 years, absence of clinical heart failure diagnosis or other known medical conditions, and negative result of a serological test for Chagas disease.

Heart tissue samples were obtained from 17 patients submitted to heart transplantation at the Messejana Hospital Heart Transplant Unit in Fortaleza, Brazil. Explanted cardiac tissue samples obtained from ventricles of subjects with end-stage CCC (*n* = 9) and normal control heart tissue samples (*n* = 8) were evaluated for the expression of miRNAs by RT-qPCR. Control heart tissues were chosen from biopsies of donor hearts without any abnormal histological findings.

### 4.3. MiRNA Selection

Based on the literature review regarding miRNAs previously associated with heart failure, cardiac fibrosis, and hypertrophy, six miRNAs were selected for analysis: miR-19a-3p, miR-21-5p, miR-29b-3p, miR-30a-5p, miR-199a-5p, and miR-208a-3p. Table 3 summarizes the most relevant altered circulating microRNAs reported in cardiovascular diseases. We used miRNA-specific forward and reverse primers with LNA technology miRCURY LNA miRNA PCR Assay (Exiqon, Copenhagen, Denmark).

### 4.4. Total RNA, MicroRNA Extraction and cDNA Synthesis

Exosomes were isolated from serum and plasma samples using the miRCURY™ Exosome Isolation Kit (Exiqon) and 0.5 mL serum/plasma from Chagas disease subjects and healthy controls, following the manufacturer’s instructions. MicroRNA was isolated from exosome preparations, cells, or heart tissue using the miRCURY ™ RNA Isolation Kit (Exiqon). Synthesis of cDNA was performed using the Universal cDNA Synthesis kit (Exiqon). Total RNA was extracted using Trizol® (ThermoFisher Scientific, Waltham, MA, USA) and cDNA was synthesized using High-Capacity cDNA Reverse Transcription Kit (ThermoFisher Scientific).

### 4.5. Quantitative Real-time PCR (RT-qPCR)

The expression levels of microRNAs were evaluated in serum and plasma samples. Quantitative real-time PCR assays were performed with miRCURY LNA™ Universal RT microRNA PCR with SYBR® and LNA™ primers set (Exiqon). Five micro RNAs (miR-16, miR-93, miR-423, cel-miR-39-3p, and SNORD44) were selected from the literature [58,59] and used for normalization upon demonstration of stable expression among the samples. Spike-in RNAs were used to calibrate the results (Exiqon).

The expression levels of *COL1A1* and *NPPB* were also evaluated by RT-qPCR. *COL1A1* expression was evaluated using SYBR™ Green PCR Master Mix (ThermoFisher Scientific) with the following primers: (forward 5’-GTGCGATGACGTGATCTGTGA–3’; reverse 5’-CGGTGGTTTCTTGGTCGGT-3’). *GAPDH* was used for normalization (forward 5’-ACAACTTTGGTATCGTGGAAGG-3’; reverse 5’-GCCATCACGCCACAGTTTC-3’). *NPPB* mRNA amplification was conducted using the Taqman™ Universal PCR Master mix and Taqman probe Hs00173590_m1. Two references genes were tested for normalization: *GAPDH* (Hs02786624_g1) and *HPRT* (Hs02800695_m1) (ThermoFisher Scientific).

### 4.6. Functional in vitro Assays with Human Cardiac Fibroblasts

Cardiac fibroblasts were isolated from myocardial surgical specimens, as previously described [28,60]. The cells were characterized by immunostaining following overnight incubation at 4 °C with the primary antibodies: α-SMA (1:100) (Dako, Glostrupe, Denmark), Vimentin (1:100) (Santa Cruz Biotechnology), DDR2 (1:100) (Abcam, Cambridge, UK), and type I collagen antibody (1:100) (Santa Cruz Biotechnology). Next, secondary antibodies and probes were used, with 1 h incubation at room temperature: Phalloidin 633 (ThermoFisher Scientific), anti-mouse IgG Alexa fluor 488-conjugated or anti-goat IgG Alexa fluor 488-conjugated, both diluted at 1:800 (ThermoFisher Scientific). The staining was performed with 4,6-diamidino-2-phenylindole (DAPI) (Vector Laboratories, Burlingame, CA, USA). The A1+ confocal microscope (Nikon, Tokyo, Japan) was used for analysis.

Cardiac fibroblasts were incubated with 10 ng/mL recombinant human TGF-β1 (Peprotech, Rocky Hill, NJ, USA) for 24 h at 37 °C incubation and 5% CO_2_ for fibroblast activation. Type I collagen (*COL1A1)* gene expression was evaluated by RT-qPCR, as described above. Protein expression was analyzed with the Operetta High Content Screening, using the Harmony high-content analysis software (Perkin Elmer, Waltham, MA, USA).

### 4.7. Modeling Human Cardiac Hypertrophy in hiPSC-CM

Two human induced pluripotent stem cell (hiPSC) lines obtained from healthy male donors were used in this study. The cells were previously generated from the reprogramming of somatic cells (skin fibroblasts and peripheral blood mononuclear cells) with a non-integrating cell reprogramming method, as previously described [61]. HiPSCs were maintained on plates coated with Matrigel (Corning, New York, NY, USA) in Essential 8 medium (ThermoFisher Scientific), in a humidified atmosphere containing 5% CO_2_ and at 37 °C. Cardiomyocyte differentiation was performed using PSC Cardiomyocyte Differentiation Kit (ThermoFisher Scientific).

The characterization of hiPSC-derived cardiomyocytes (hiPSC-CM) was performed by immunofluorescence and FACS analyses. For immunofluorescence, the cells were fixed with 4% paraformaldehyde (PFA), washed with PBS and permeabilized with 0.05% Triton solution. The following primary antibodies and dilutions were used, with overnight incubation at 4 °C: alpha-actinin (Sigma-Aldrich, St. Louis, MI, USA; 1:100), MF20 (DSHB, Iowa City, IA; 1:100), anti-cTNT (ThermoFisher Scientific; 1:100) GATA-4 (Santa Cruz Biotechnology, Dallas, TX, USA; 1:100). The cells were incubated with the secondary antibodies, for 1 h at room temperature with anti-mouse IgG Alexa fluor 568-conjugated or anti-rabbit IgG Alexa fluor 488-conjugated, both diluted at 1:500 (ThermoFisher Scientific). Nuclei were stained with DAPI (Vector Labs, Burlingame, CA, USA) and the cells were examined at a confocal microscope (FluoView 1000, Olympus, Tokyo, Japan). For FACS analysis, the cells were dissociated with trypsin-EDTA solution (ThermoFisher Scientific), fixed, permeabilized (Cytofix/Cytoperm, BD Biosciences, Franklin Lakes, NJ, USA), and stained for cardiac troponin with anti-cTNT (ThermoFisher Scientific) for 1 h, followed by incubation with a secondary antibody conjugated with Alexa Fluor 633 (ThermoFisher Scientific). Data acquisition was performed using the LSR Fortessa flow cytometer (BD Biosciences), and analysis was performed using the FACS DIVA v. 6.3 software.

For functional analysis, a hypertrophic response was induced in hiPSC-CM by incubation with 10 nM endothelin-1 (ET-1, Sigma-Aldrich), as previously described [30]. Cell-free culture supernatant was collected 24 h later for N-terminal prohormone of brain natriuretic peptide (NT-pro-BNP) measurement (Vidas® NT-pro-BNP, Biomerieux, Marcy, France). Additional wells were stained with anti-cTNT for cell size measurements in the Operetta High Content Screening (Perkin Elmer), using the Harmony High-Content Analysis software (Perkin Elmer).

### 4.8. Statistical Analyses

Categorical data were presented as numbers (percentages), and continuous data were expressed as mean (SD) or median (interquartile range). Comparisons of continuous variables among groups were performed with analysis of variance (ANOVA) test or Kruskal-Wallis, depending on normality assessed by the Shapiro-Wilk test. Chi-Square or Fisher tests were applied for proportion comparisons. Correlations between continuous variables were evaluated by Pearson or Spearman coefficients. Analyses of candidate microRNAs and clinical parameters were performed using Statistic Data Analysis STATA^®^ and *p* < 0.05 (two-tailed) was considered statistically significant.

RT-qPCT data analysis was performed using Exiqon GenEx v6 (Exiqon) software and Threshold Cycle Method [62]. Expression levels were logarithmically transformed for statistical analysis using Graphpad Prism v6 (2015).

## Figures and Tables

**Table 1:** Clinical and demographic parameters of chronic Chagas disease subjects.

Variables	Indeterminate Form (*n* = 10)	CCC (*n* = 28)
Age (years)	59 ± 8	60 ± 7
Male gender	7 (70%)	18 (65%)
% fibrosis (CMR)	3.1 ± 5	19 ± 15
EF (CMR)	77.5 ± 13	32 ± 10
Strain (LV)	22.5 ± 6	10 ± 4
Ventricular arrhythmia	8 (80%)	28 (100%)
SV arrythmia	8 (80%)	23 (82%)
HR variability	123 ± 22	120 ± 39

CMR = Cardiovascular magnetic resonance. EF = Ejection fraction. LV = left ventricular. SV = Supraventricular. HR = Heart rate.

**Table 2:** Correlation of microRNA expression and clinical parameters in chronic Chagas disease subjects.

miRNAs	Cardiac Dysfunction	Age	NYHA (II-IV)	%Fibrosis	EF (CMR)	Strain (LV)	Ventricular Arrhythmia	SV Arrhythmia	HR Variability
miR-19a-3p	*r* = 0.47	*r* = 0.20	*r* = 0.47	*r* = 0.41	*r* = −0.40	*r* = −0.36	*r* = 0.15	*r* = 0.09	*r* = −0.11
	*p* = 0.003 **	*p* = 0.22	*p* = 0.003 **	*p* = 0.01 *	*p* = 0.01 *	*p* = 0.02 *	*p* = 0.37	*p* = 0.59	*p* = 0.54
miR-21-5p	*r* = 0.42	*r* = 0.06	*r* = 0.42	*r* = 0.38	*r* = −0.40	*r* = −0.34	*r* = 0.16	*r* = 0.02	*r* = −0.21
	*p* = 0.008 **	*p* = 0.70	*p* = 0.008 **	*p* = 0.03 *	*p* = 0.01 *	*p* = 0.04 *	*p* = 0.32	*p* = 0.89	*p* = 0.24
miR-29b-3p	*r* = 0.47	*r* = 0.04	*r* = 0.47	*r* = 0.36	*r* = −0.34	*r* = −0.32	*r* = 0.24	*r* = 0.27	*r* = −0.34
	*p* = 0.02 *	*p* = 0.79	*p* = 0.02 *	*p* = 0.03 *	*p* = 0.03 *	*p* = 0.05	*p* = 0.13	*p* = 0.09	*p* = 0.05
miR-199b-3p	*r* = 0.24	*r* = −0.27	*r* = 0.24	*r* = 0.01	*r* = −0.28	*r* = −0.18	*r* = 0.03	*r* = −0.07	*r* = −0.11
	*p* = 0.16	*p* = 0.12	*p* = 0.16	*p* = 0.96	*p* = 0.10	*p* = 0.31	*p* = 0.85	*p* = 0.66	*p* = 0.54

*n* = 38 subjects (indeterminate form and chronic Chagas cardiomyopathy). EF = ejection fraction. CMR = cardiovascular magnetic resonance. LV = left ventricular. SV = supra ventricular, HR= heart rate. Spearman, * *p* < 0.05; ** *p* < 0.01.

**Table 3:** Circulating microRNA expression levels in cardiovascular disease.

microRNA	Disease	Sample	Regulation	References
miR-19a	Heart failure	plasma	upregulated	[18]
miR-21	Aortic stenosis	plasma	upregulated	[19]
miR-21	Acute coronary syndrome	plasma	upregulated	[51]
miR-21	Chronic cardiovascular disease (atherosclerosis)	peripheral blood	upregulated	[49]
miR-21	Congestive heart failure	serum	upregulated	[17]
miR-29	Hypertrophy cardiomyopathy	plasma	upregulated	[20]
miR-30a	Acute myocardial infarction	plasma	upregulated	[52]
miR-30a	Ischemic stroke	plasma	upregulated	[53]
miR-30a	Chronic heart failure	serum	upregulated	[54]
miR-30a	Hypertrophy cardiomyopathy	plasma	upregulated	[20]
miR-199	Hypertrophy cardiomyopathy	plasma	upregulated	[20]
miR-208a	Coronary artery disease	plasma	upregulated	[55]
miR-208a	Acute myocardial infarction (TASH: transcoronary ablation of septal hypertrophy)	serum	upregulated	[56]
miR-208	Acute myocardial infarction	plasma	upregulated	[57]
miR-208a	Chronic Chagas disease	plasma	upregulated	[25]

## Supplementary Materials

Supplementary materials can be found at https://www.mdpi.com/1422-0067/20/16/4064/s1.
