# Supplementary material for: Circulating miRNAs as Potential Biomarkers Associated with Cardiac Remodeling and Fibrosis in Chagas Disease Cardiomyopathy
Source: Int J Mol Sci. 2019 Aug 20;20(16):4064. doi: 10.3390/ijms20164064 (PMC6721092; doi:10.3390/ijms20164064)
Supplement: Supplementary file 1 [file ijms-20-04064-s001.pdf]

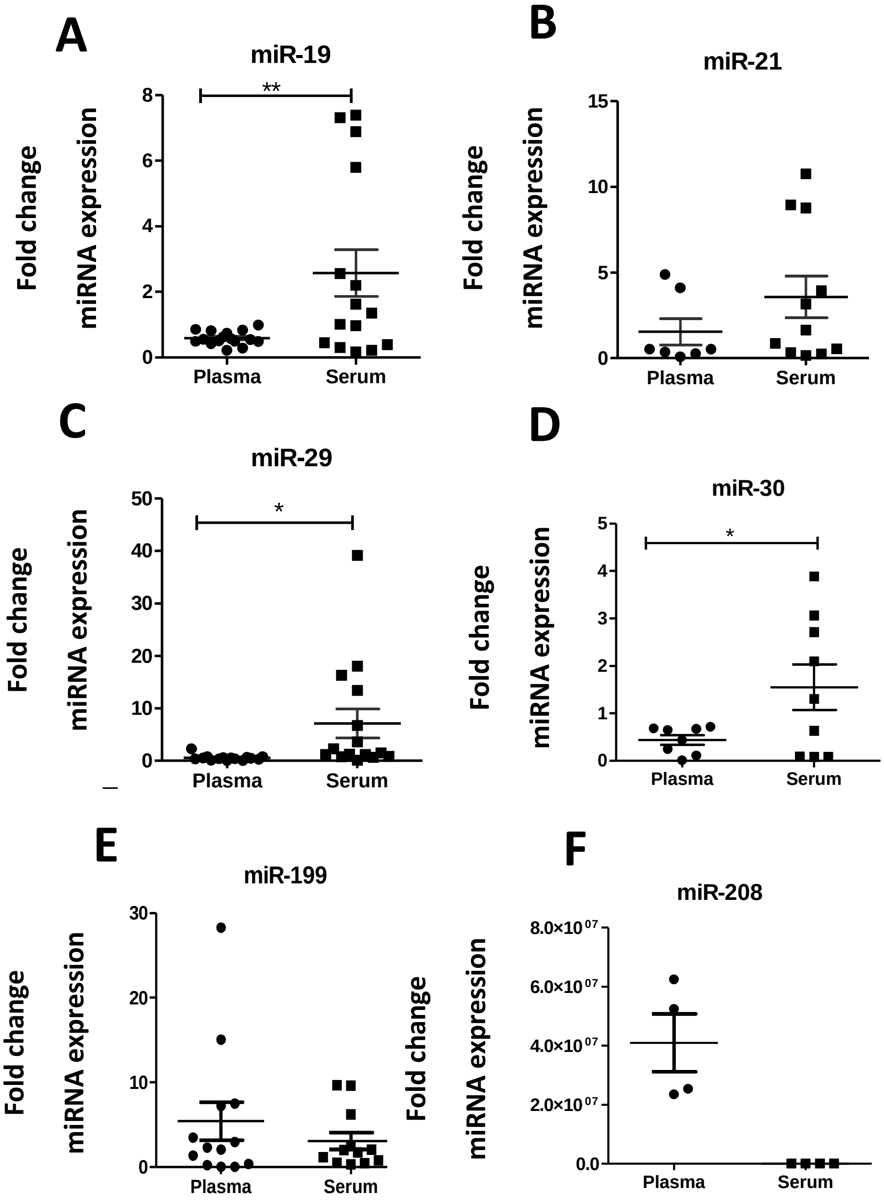

Figure S1. Comparison of circulating miRNA expression in serum versus plasma sample by RT-qPCR (**A**–**F**). Serum and plasma samples from 16 chronic Chagas disease subjects by RT-qPCR. Student's *t*-test. * $p < 0.05$; ** $p < 0.001$.
